# Total joint replacement with the stock Biomet system in adult hemifacial microsomia without glenoid fossa: a case report and literature review

**DOI:** 10.1186/s40902-025-00478-5

**Published:** 2025-10-15

**Authors:** Kang Hee Yu, Jeong Joon Han, Soon Jung Hwang

**Affiliations:** 1 Department of Dentistry, School of Medicine, Chungbuk National University, Cheongju-si, Korea, Republic of; 2 Department of Oral and Maxillofacial Surgery, Chungbuk National University Hospital, Cheongju-si, Korea, Republic of; 3 Department of Oral and Maxillofacial Surgery, Seoul National University Dental Hospital, Seoul, Korea, Republic of; 4 Department of Oral and Maxillofacial Surgery, Dental Research Institute, School of Dentistry, Seoul National University, Seoul, Korea, Republic of; 5 HSJ Dental Clinic for Oral and Maxillofacial Surgery, Seoul, Korea, Republic of

**Keywords:** Hemifacial macrosomia, Zygomatic arch absence, Glenoid fossa reconstruction, Autologous bone graft, Stock total joint replacement, Frontal ramal angle

## Abstract

**Background:**

In patients with hemifacial microsomia accompanied by mandibular fossa deficiency and severe atrophy of the mandibular ramus, customized total joint replacement (TJR) is commonly used to restore masticatory and joint function and improve facial asymmetry. However, in countries where customized TJR is not approved, or for patients for whom the cost is prohibitive, a stock TJR system must be considered. In cases with a severely medially inclined frontal ramal angle and a lack of supporting bone for the fossa component, using a stock TJR poses significant technical challenges.

**Case presentation:**

This case report describes the use of autogenous bone grafting to overcome these limitations. An 18-year-old male with HFM type IIB on the left side received staged procedures, including bone grafting between mandibular proximal and distal segments to increase the frontal ramal angle, autogenous reconstruction of the mandibular fossa to enable fixation of the TJR fossa component, and orthognathic surgery.

**Conclusions:**

The frontal ramal angle improved by approximately 6.5 degrees, and 12.42 mm advancement of the pogonion could be achieved in lateral cephalogram. Successful functional and esthetic outcomes were achieved, with stable maintenance of a stock Biomet TJR over a seven-year period.

## Background

Hemifacial microsomia (HFM) is the most common congenital disorder of the face after cleft lip and palate [1]. The cause of HFM remains unknown. The most widely used classification system is Kaban’s modification of the Pruzansky classification system [2]. In severe forms, such as type IIB (condylar dysplasia or absence of the condyle with a flat or absent glenoid fossa) and type III (complete absence of the ramus and fossa), temporomandibular joint (TMJ) function and facial symmetry are severely compromised.

In patients with HFM, functional and aesthetic improvements can be achieved through mandibular alloplastic joint reconstruction. However, in cases with type IIB or III, where the zygomatic arch is absent and the ramus is severely displaced medially due to ramal dysplasia, a stock total joint replacement (TJR) system cannot be applied because fixation of the fossa component of the alloplastic joint is not feasible and correction of mandibular asymmetry in terms of a symmetrical frontal ramal angle is not possible. Reconstruction of a substantial glenoid fossa with bone grafts presents a significant challenge. Additionally, due to markedly insufficient thickness and severe displacement with inadequate lateral inclination of the mandibular body and ramus in HFM patients, it is difficult to position the alloplastic joint to closely match the frontal ramal angle of the non-affected side. Severe mandibular asymmetry remains after orthognathic surgery because the height and volume of the mandibular body on the affected side are smaller compared to those on the non-affected side [3].

To overcome those limitations, customized TJR or extended customized TJR has been applied and successful results have been reported [4, 5]. However, there are countries where these types of TJR systems are not available to patients, because they are not approved by the national Food and Drug Administration (FDA). Moreover, the high cost of these systems is a great limitation for the application for many patients, especially when not covered by medical insurance.

In this report, we present a surgical strategy to overcome these limitations. Autologous bone grafting was performed to reconstruct the area required for fixation of the fossa component, which was followed by successful TJR with a stock Biomet system (Zimmer Biomet Holdings®, Warsaw, U.S.). Furthermore, on the affected side, a sagittal split ramus osteotomy(SSRO) was carried out, and an iliac bone graft was interposed between the proximal and distal segments to recreate a frontal ramal angle similar to that of the non-affected side. Through this approach, we were able to achieve not only functional rehabilitation of TMJ but also significant aesthetic improvement, and the TJR was successfully maintained over a period of seven years.

## Case presentation

**Fig. 1:**
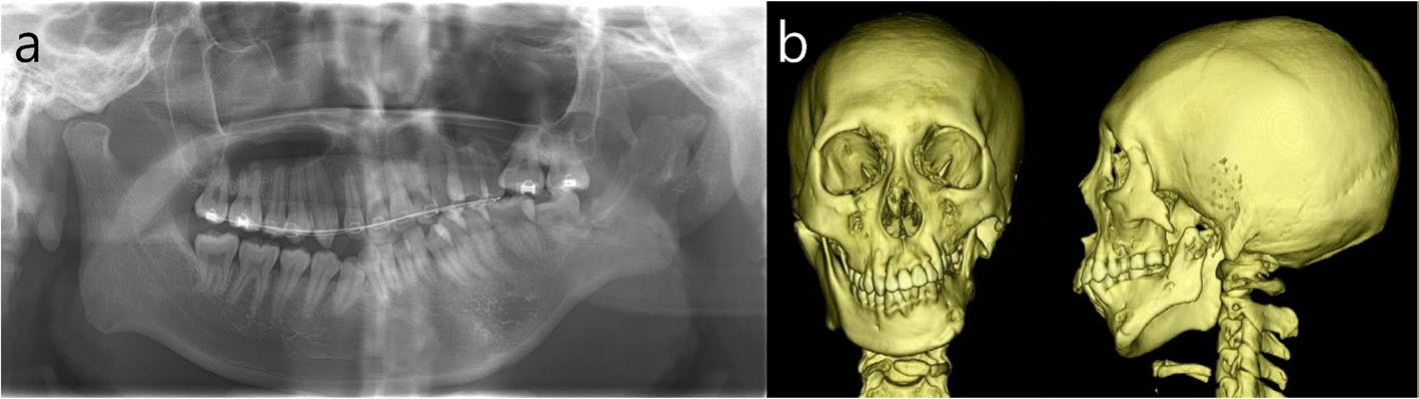
Radiographs on initial visit. **a** Panoramic view. **b** frontal and lateral views of 3D CT

This study was approved by the Institutional Review Board of Seoul National University Dental Hospital (ERI 25024). An 18-year-old male with HFM type IIB on the left side visited for consultation regarding TMJ reconstruction and correction of mandibular asymmetry. He was previously treated at another hospital with iliac bone graft for augmentation of left mandibular body and rib cartilage graft for reconstruction of left ear. The grafted iliac bone was nearly completely resorbed. Problem lists of the patient on the left facial side were absence of glenoid fossa and posterior zygomatic arch, absence of condyle, atrophied short mandibular body, severe maxillary canting, thin mandibular ascending ramus, small frontal ramal inclination (medially inclined ramus), and mandibular retrognathism with asymmetry (Fig. 1). The stepwise surgical procedures for the improvement of those problems were as follows (Table 1).

**Table 1:** Sequence of surgical steps

The order of surgery	The surgical procedure
The first surgery	DO of the left maxilla and mandible
The second surgery	Iliac bone graft to the left mandibular ascending ramus, Reconstruction of the left mandibular fossa with ramal bone graft
The third surgery	Le fort I osteotomy, SSRO on the right mandible, TJR on the left mandible, Genioplasty
The fourth surgery	Plate removal, Mandibuloplasty, Genioplasty, Angle reduction on the right mandible

**Fig. 2:**
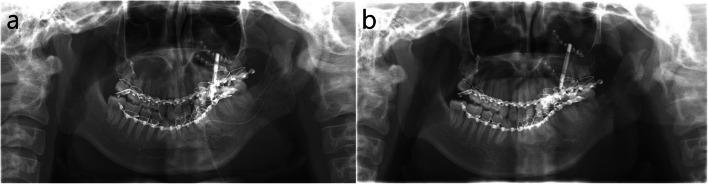
Panoramic view after the first surgery. **a** Immediate after surgery for DO device installation at maxilla and mandibular SSRO on the left side. **b** 3 weeks after DO

Distraction osteogenesis (DO) of the left maxilla via Le Fort I osteotomy and of the mandible by unilateral SSRO for the correction of maxillary and mandibular canting on the left side was conducted. After Le Fort I osteotomy, a thin 2-hole miniplate was installed on the zygomaticomaxillary buttress on the right side, which acted as a rotation center during canting correction by DO, and a DO device was installed on the left maxilla (Fig. 2a). After mandibular osteotomy with SSRO on left mandible, intermaxillary fixation was done and the planned distraction movement of left maxilla and mandibular distal segment was tested. One week after surgery, DO was started at 1 mm per day for seven days, until maxillary canting was corrected (total 7 mm DO) (Fig. 2b).

**Fig. 3:**
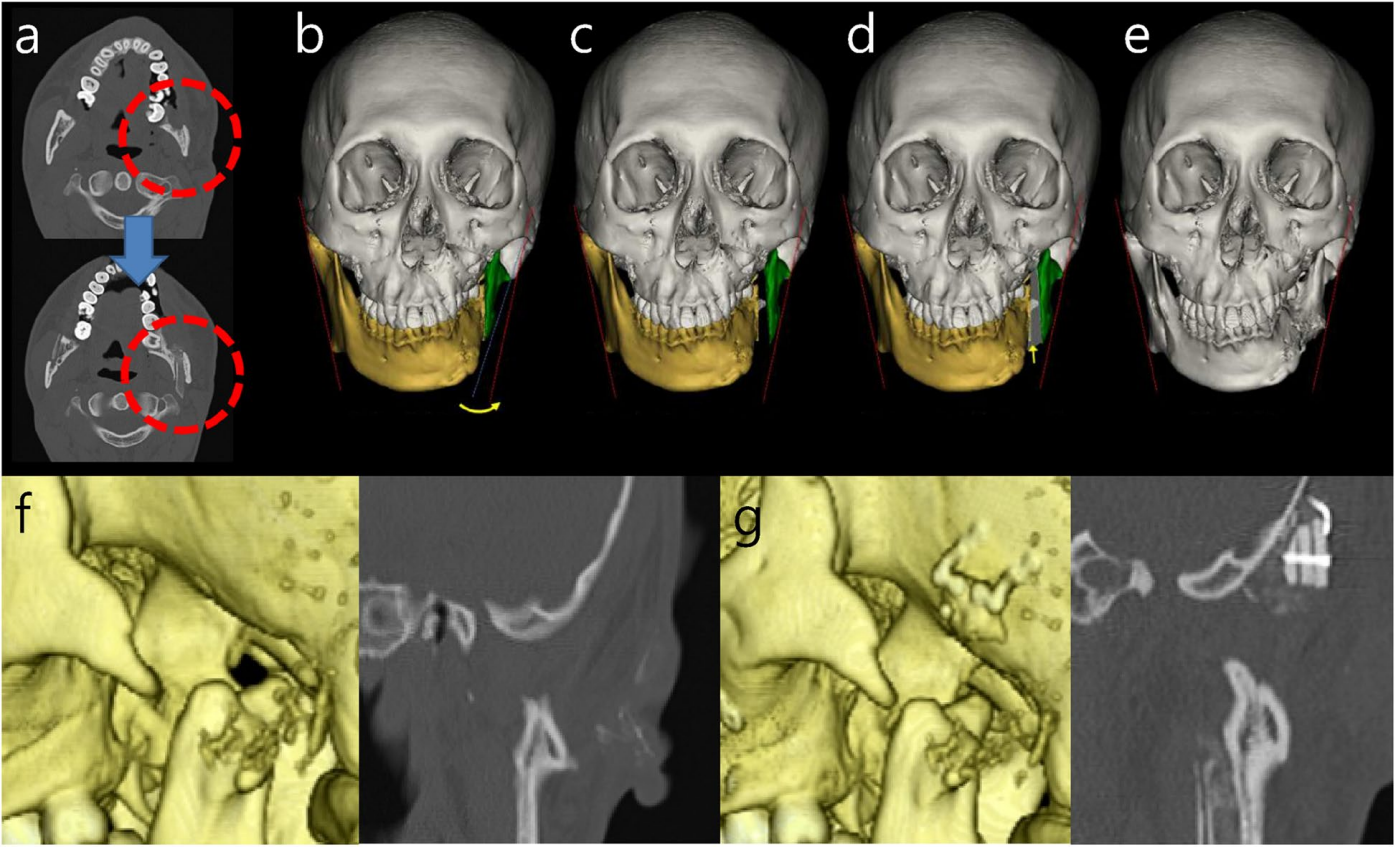
CT views before and after the second surgery. **a** Axial cut of CT before and after autogenous intersegmental bone graft after SSRO at left mandibular ramus, **b** simulation SSRO on the left side (red line – symmetrical frontal ramal inclination on right and left side), **c** simulation of lateral swing movement of the left proximal segment after SSRO, **d** simulation of intersegmental bone graft, **e** 3D CT image after the second surgery, **f** initial status of temporal bone; absence of glenoid fossa in 3D CT (left) and coronal cut of CT (right), **g** reconstructed flat osseous structure on the temporal slope by ramal bone graft for the installation of mandibular fossa part of stock Biomet TJR in 3D CT (left) and coronal cut of CT (right)

Six months after the first surgery, autogenous bone grafting was performed for the correction of the thin and medially inclined left mandibular ascending ramus and the absence of the glenoid fossa on the left side. Unilateral SSRO was performed and an autogenous iliac bone graft was positioned between the proximal and distal segments to increase the ramal thickness and decrease the medial inclination of the left mandibular ramus, which enabled installation of the mandibular part of the TJR with adequate frontal ramal inclination and mandibular symmetry in the frontal view. Through this procedure, an improvement of approximately 6.5 degrees in the left frontal ramal angle was achieved (Fig. 3a ~ e). Because of the absence of the mandibular fossa (Fig. 3f), it was necessary to reconstruct a horizontally flat bony structure on the temporal bone for installation of the fossa part of TJR. Ramal bone was harvested from the right side, and three pieces of ramal bone were layered and fixed in a pyramidal form with a mini screw to adapt to the temporal slope. Then, it was stabilized with two mid-plates on the temporal bone. The dead space between the bone block and temporal bone was filled with an alloplastic bone substitute soaked with 0.5 cc recombinant human bone morphogenetic protein 2 (rhBMP-2) (Novosis®Dent, CG Bio, Seoul, Republic of Korea) (Fig. 3g).

**Fig. 4:**
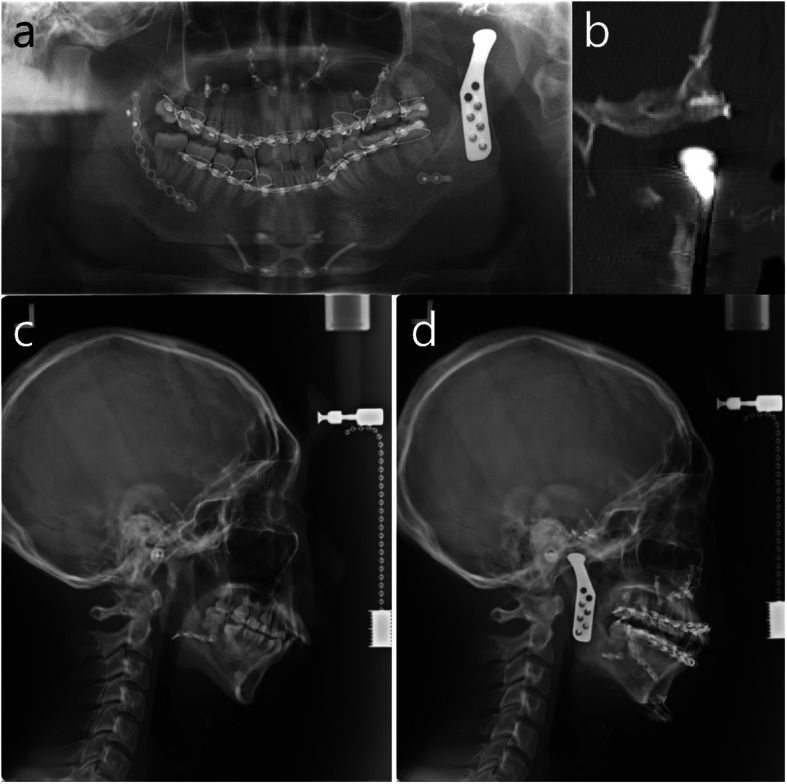
Radiographs after TJR and two jaw surgery in the third surgery. **a** panoramic view: TJR on the left side, **b** coronal cut of CT at TMJ. Installed fossa part of TJR on the well regenerated bone graft at temporal bone and mandibular part of TJR, **c** lateral cephalogram before two jaw surgery and TJR, **d** lateral cephalogram after two jaw surgery and TJR

**Fig. 5:**
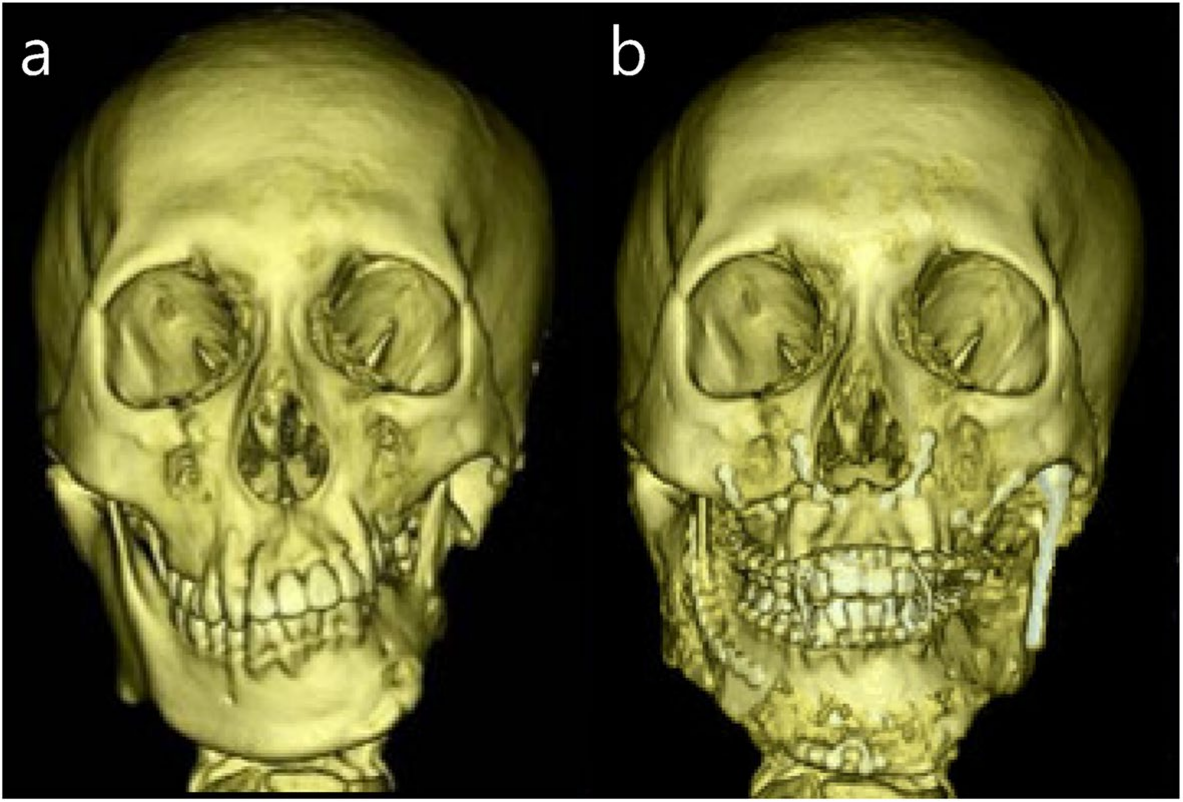
Frontal view of 3D CT before and after two jaw surgery and TJR. **a** initial visit, **b** 6 months after two jaw surgery and TJR

**Fig. 6:**
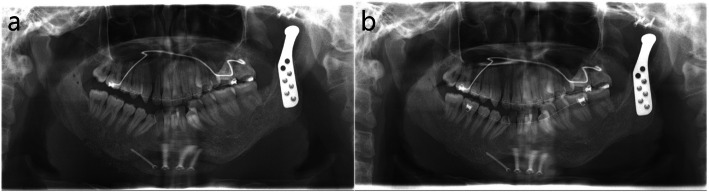
Panorama views after mandibular contouring surgery in the fourth surgery. **a** Immediate after the fourth surgery, **b** 3 months after the fourth surgery

Eight months after the second surgery, transplanted autogenous bone grafts at the left mandible and left temporal bone were well fused and stabilized. Orthognathic surgery with Le Fort I osteotomy, unilateral SSRO on the right side, and genioplasty combined with TJR with a stock Biomet system on the left side for the reconstruction of the left TMJ was performed for the correction of mandibular retrognathism with asymmetry. After intermaxillary fixation with final wafer, the positions of these two parts were regulated, so that the relationship between the fossa part and the condylar part was appropriate. And the bone surface was trimmed to maximize contact between the prosthesis and the bone. First, mandibular fossa part was fixed with two screws and then condylar part was manually guided to the fossa part and fixed with two screws on the mandibular ramus. Intraoral checking for occlusion and condylar position was done after the removal of intermaxillary fixation, and intermaxillary fixation with the final wafer was conducted. The surgery of TJR was finished with additional fixation on the fossa part with two screws and on the condylar part with three screws. Postoperative cephalometric changes were 0.13°- increase in SNA, 4.03°- decrease in SNB, 6.59°- decrease in the mandibular plane angle, and 12.42 mm advancement of the pogonion (Figs. 4 and 5). Plate removal and mandibuloplasty, including genioplasty, right angle reduction, and bone graft for the correction of residual asymmetry, were performed five months following the third surgical procedure (Fig. 6a).

Maxillary canting was successfully corrected by 7 mm DO. Maxillary and mandibular length were well elongated as expected. However, the left mandibular proximal segment lacked joint structures, including the capsule and ligaments, thus providing insufficient resistance against simultaneous downward movement of the distal segment during DO. It moved downward together with the distal segment during DO, which was evident by the absence of a bone step at the antegonial notch. In the second surgery, thin and medially positioned left mandibular ramus was changed to a thicker and symmetrical form, thus providing adequate structure for TJR. In addition, the osseous structure for the installation of the fossa component of the stock Biomet TJR was successfully reconstructed by the ramal bone graft combined with an alloplastic bone substitute soaked with rhBMP-2. Thanks to these preparatory surgical treatments, the main operations with TJR and orthognathic surgery could be conducted with successful functional and aesthetic outcomes. TMJ function was normalized by TJR, and skeletal malocclusion with facial asymmetry could be easily and effectively corrected according to the surgical plan. After the last surgery, including facial contouring with additional genioplasty, right angle reduction, and bone graft, balanced facial symmetry was achieved (Fig. 6), and stable occlusion was maintained during postoperative orthodontic treatment. The TJR was well maintained for seven years.

## Discussion

The surgical needs in patients with HFM depend entirely on the type and severity of the facial abnormalities. Surgical interventions are designed to restore the patient’s craniofacial form and function and must account for the expected facial growth pattern, timing of dental eruption, schedules for school and extracurricular activities, along with other psychosocial factors. In general, surgical treatments for HFM include bone grafting, free flaps, DO, TJR, and orthognathic surgery [6].

TMJ and glenoid fossa can be reconstructed with alloplastic materials in cases with large defects which would make the procedure difficult with autogenous bone grafting [7]. Long-term follow-up of the effects of the Christensen Fossa Eminence Prosthesis on the mandibular condyle has not been widely reported. In a study by Chase et al., treatment with a fossa eminence prosthesis resulted in significant functional improvement and pain reduction [8]. The patients in their study had diagnoses of osteoarthritis, rheumatoid arthritis, or internal derangement associated with their TMJ.

Securing an autogenous glenoid fossa to an abnormally sloped, canted temporal bone lacking a bony flange is particularly difficult. Additionally, recreating a broad, stable articular surface using autogenous materials remains technically demanding [9]. Reconstruction of the glenoid fossa using costochondral grafts has been explored in previous studies, which presented a technique of immediate reconstruction of the petrous portion of the temporal bone and of the mandible [10]. Richter et al. reconstructed the cranial base and condyle during the same operation using calvarial bone [11]. The reconstructed cranial base must protect the dura and brain from the significant forces generated by the masticatory muscles, for which calvarial bone is particularly suitable due to its high density and lower resorption rate compared to iliac bone grafts [12]. In addition, as it is composed of cortical bone, calvarial bone provides firm anchorage for screw fixation, allowing immediate stability that cannot be achieved with costochondral grafts.

In our present study with HFM IIB subgroup, an intersegmental autogenous bone graft between the proximal and distal segments after SSRO was performed, which resulted in improvement of mandibular asymmetry. Not only was the thin and medially positioned left mandibular ramus changed to a thicker and symmetrical form, but also it enabled adequate frontal angulation of the mandibular part of the TJR. In addition, flat bone structure at the temporal bone for the installation of fossa part of TJR could be easily reconstructed by mandibular ramal bone and alloplastic bone substitute soaked with rhBMP-2. In situations with regulatory or financial constraints on customized TJR, our surgical approaches can be practically utilized for an alternative method.

Given the increasing use of custom-made extended TMJ prostheses and the absence of a standardized classification, a classification system based on mandibular and fossa extension patterns was proposed. A two-part classification system for extended TJR was developed to categorize prosthetic designs according to mandibular and skull base involvement, with the goal of enhancing clarity in surgical planning and design specification [13].

This study is a single case report, thus, the results may not be generalizable to all patients with hemifacial microsomia without glenoid fossa. The lack of a larger sample size limits the ability to draw broader conclusions regarding the efficacy and applicability of the stock Biomet TJR system in this patient population. Further study with a larger cohort is needed to validate the outcomes observed in this case and to better understand the potential for broader clinical application.

## Conclusion

Patients with HFM without a condyle and glenoid fossa of the temporal bone have severe limitations for the use of the stock system of TJR. Even though the extended custom systems of TJR are increasingly applied for such types, these systems are not available for many patients due to regulatory and financial constraints. In the present report, the osseous structure for the installation of the fossa component of the stock Biomet TJR was successfully reconstructed by the ramal bone graft combined with an alloplastic bone substitute soaked with rhBMP-2, and TJR could be well maintained for a long time. Moreover, an intersegmental autogenous bone graft at the mandibular ascending ramus after SSRO enabled adequate frontal angulation of the mandibular part of the TJR with the improvement of mandibular asymmetry. This staged approach offers a practical and viable alternative for patients in environments where customized TJR systems are not feasible, making it a valuable contribution to this field.

## Data Availability

No datasets were generated or analysed during the current study.

## Abbreviations

TJR: Total joint replacement
HFM: Hemifacial microsomia
TMJ: Temporomandibular joint
FDA: Food and Drug Administration
SSRO: Sagittal split ramus osteotomy
DO: Distraction osteogenesis
